# Porphyria cutanea tarda and patterns of long-term sick leave and disability pension: a 24-year nationwide matched-cohort study

**DOI:** 10.1186/s13023-022-02201-3

**Published:** 2022-02-22

**Authors:** Carl Michael Baravelli, Aasne Karine Aarsand, Sverre Sandberg, Mette Christophersen Tollånes

**Affiliations:** 1 Department of Medical Biochemistry and Pharmacology, Haukeland University Hospital, Norwegian Porphyria Centre (NAPOS), P. O. Box 1400, 5021 Bergen, Norway; 2 Department of Disease Burden, Norwegian Institute of Public Health, Bergen, Norway; 3 Norwegian Institute of Public Health, Bergen, Norway; 4 Norwegian Organisation for Quality Improvement of Laboratory Examinations (NOKLUS), Haraldsplass Deaconess Hospital, Bergen, Norway; 5 Department of Global Public Health and Primary Care, University of Bergen, Bergen, Norway

**Keywords:** Porphyria cutanea tarda, Long-term sick leave, Sick leave absences, Disability pension

## Abstract

**Background:**

Porphyria cutanea tarda (PCT) is a skin disorder caused by a defect in the liver enzyme uroporphyrinogen decarboxylase and is associated with hepatitis C virus infection, high alcohol intake, smoking and iron overload. Data on the long-term morbidity of PCT is lacking.

**Methods:**

We conducted a nationwide matched cohort study over a 24-year period. The study sample included 534 persons aged 18–67 years with a biochemically confirmed PCT diagnosis and a sample of 21,360 persons randomly selected from the working age population, matched on age, sex and educational attainment. We investigated if persons with sporadic and familial PCT had an increased risk of long-term sick leave (LTSL) or disability pension. We further assessed risk before (pre-PCT), during (during-PCT) and after (post-PCT) the typical period of first onset to diagnosis, treatment and remission.

**Results:**

Overall, persons with PCT had a 40% increased risk (hazard ratio [HR] = 1.4, 95% confidence interval [CI] = 1.3, 1.5) of LTSL and a 50% increased risk (HR = 1.5, CI = 1.3, 1.7) of disability pension. Risk of disability pension was increased pre-PCT (HR = 1.3, CI 1.3 (1.0, 1.6), during-PCT (HR 1.5, CI 1.0, 2.2) and post-PCT (HR = 2.0, CI 1.5, 2.6). For LTSL, risk was increased pre-PCT (HR = 1.3, CI 1.1, 1.4) and during-PCT (HR = 1.5, CI 1.1, 2.1), but not post-PCT. Risk was greatest in persons with sporadic than familial PCT. Diagnostic reasons for disability pension that were increased compared to matched controls were PCT or skin disease in 11 of 199 cases (PCT: n = 7, incident rate ratios [IRR] = 49.2, CI = 38.8, 62.4; diseases of the skin and subcutaneous tissue, n = 4, IRR = 4.2, CI = 1.6, 11.0). The vast majority of diagnostic reasons for accessing disability pension were related to comorbidities, PCT susceptibility factors and more general health issues such as: malignant neoplasms (n = 12, IRR = 2.4, CI = 1.4, 4.2), substance and alcohol dependence (n = 7, IRR = 5.0, CI = 2.5, 10.1), neurotic and mood—disorders (n = 21, IRR = 1.7, CI = 1.1, 2.6), and diseases of the musculoskeletal system and connective tissue (n = 71, IRR = 2.5, CI = 1.9, 3.2).

**Conclusions:**

Persons with PCT have an increased risk of LTSL and disability pension indicating significant morbidity in this patient group. Appropriate long-term follow-up and monitoring for relapses and co-morbid diseases are recommended.

**Supplementary Information:**

The online version contains supplementary material available at 10.1186/s13023-022-02201-3.

## Background

Porphyria cutanea tarda (PCT) is a hepatic and metabolic disorder caused by a defect of the hepatic enzyme uroporphyrinogen decarboxylase (UROD) [1]. In susceptible individuals, impaired UROD activity causes accumulation of porphyrins in the liver, which travel via the blood stream to the skin, where they act as photosensitisers. Symptoms arise in the form of bullae, fragile skin, hypertrichosis and hyperpigmentation in the sun-exposed areas, mostly of the hands and face [2]. Symptom debut typically peaks in the 5th decade of life, but can develop earlier, and has an approximate equal sex ratio [3]. In Norway, the incidence of PCT is estimated at 1 in 100,000, with about 50% being “acquired” (sporadic PCT) and 50% associated with a mutation in *UROD* gene (familial PCT) [3], due to two frequently occurring mutations [3]. In most other populations, the familial form makes up 20% of cases. Although familial UROD activity is reduced by up to 50 per cent in familial PCT, exposure to exogenous factors are required for clinical disease [4] and only a small number of gene mutation carriers develop symptoms [4]. The familial and sporadic types are associated with the same types of susceptibility factors; such as hepatitis C and B virus infection, high alcohol intake, smoking, iron overload including genetic hemochromatosis and the use of oestrogens [3, 5–7]. Liver damage and some degree of iron overload are typically observed [8]. Treatment includes reduced exposure to sunlight, the removal of precipitating factors, reduction of iron overload by repeated venesection and/or low dose chloroquine treatment to increase the mobilization of porphyrins from the liver [9, 10]. Such therapies usually result in prolonged remission in most patients, but patients are at risk for relapses and annual follow-ups are recommended [11, 12]. PCT is associated with an increased risk of hepatocellular carcinoma (HCC) [13–17] and diabetes mellitus [18].

Norway has a social assistance programme in which the employer pays the first 16 days of sickness absence and the social insurance scheme compensates beyond this until 250 days/52 weeks. Persons thereafter may qualify for disability pension if they are unable to return to work following this period of sickness absence, if they have a reduced earning capacity of at least 50% due to illness or injury, they have attempted treatment and rehabilitation, and are aged 18–66 years. Disability pension is paid until 67 years of age when persons transfer to age-retirement pension. Early exit of the workforce due to poor health is a burden for the individual, the workplace, society and the economy [19].

Even though PCT is a hepatic disease, it is typically seen by health care professionals as a treatable skin disorder. Patients, on the other hand, may perceive their illness as chronic and systematic and that may cause a range of health problems [20]. In a previous study, we found an increased risk of premature death in persons with sporadic PCT, although not familial PCT, due mostly to liver disease and alcohol and substance abuse [17]. However, data on long-term morbidity of PCT is generally scarce and the effect of this disease on patients’ working ability is little investigated. An increased rate of long-term sick leave (LTSL) and disability pension is a good indicator of ill health and decreased function within a patient population during their working years.

The aims of our study were: (1) to investigate if patients with PCT receive LTSL or disability pension due to their PCT diagnosis or PCT-associated comorbidities, (2) examine differences between patients who receive benefits or did not and, (3) to assess differences between persons with sporadic and familial PCT, and (4) to assess the morbidity of PCT and the effect of this disease on patients’ lives.

## Methods

### Data sources

Disbursements of LTSL and disability pension were derived from the Forløpsdatabasen-Trygd Database. The database, maintained by the Norwegian Labour and Welfare Administration and administered by Statistics Norway, contains complete records regarding payment of state benefits since 1992 [21]. The primary diagnosis for the LTSL was coded according to the International Classification for Primary Care, 2nd edition (ICPC-2) for LTSL and the International Classification for Disease, 9th (ICD-9) and 10th (ICD-10) editions, from 1992–1997 and 1998 to the present, respectively. All LTSL and disability pension diagnostic codes we investigated are presented in Additional file 1: Table S1.

Porphyria diagnoses, biochemical and clinical data was derived from the Norwegian Porphyria Registry [22], which was established in 2002 and is administered by the Norwegian Porphyria Centre [23]. All individuals with overt PCT are invited to participate [24]. At the time of study the data comprised of patient-reported questionnaires and biochemical and genetic laboratory data. The registry is based on informed consent [25].

Demographic information of all Norwegian residents was derived from The National Population Register [26]. Individual-based educational attainment was derived from the National Education Database [27]. Statics Norway, producing a de-identified research database, performed precise record linkage between the individual-level data sources in 2018.

### Study design, study population and matched controls

**Fig. 1:**
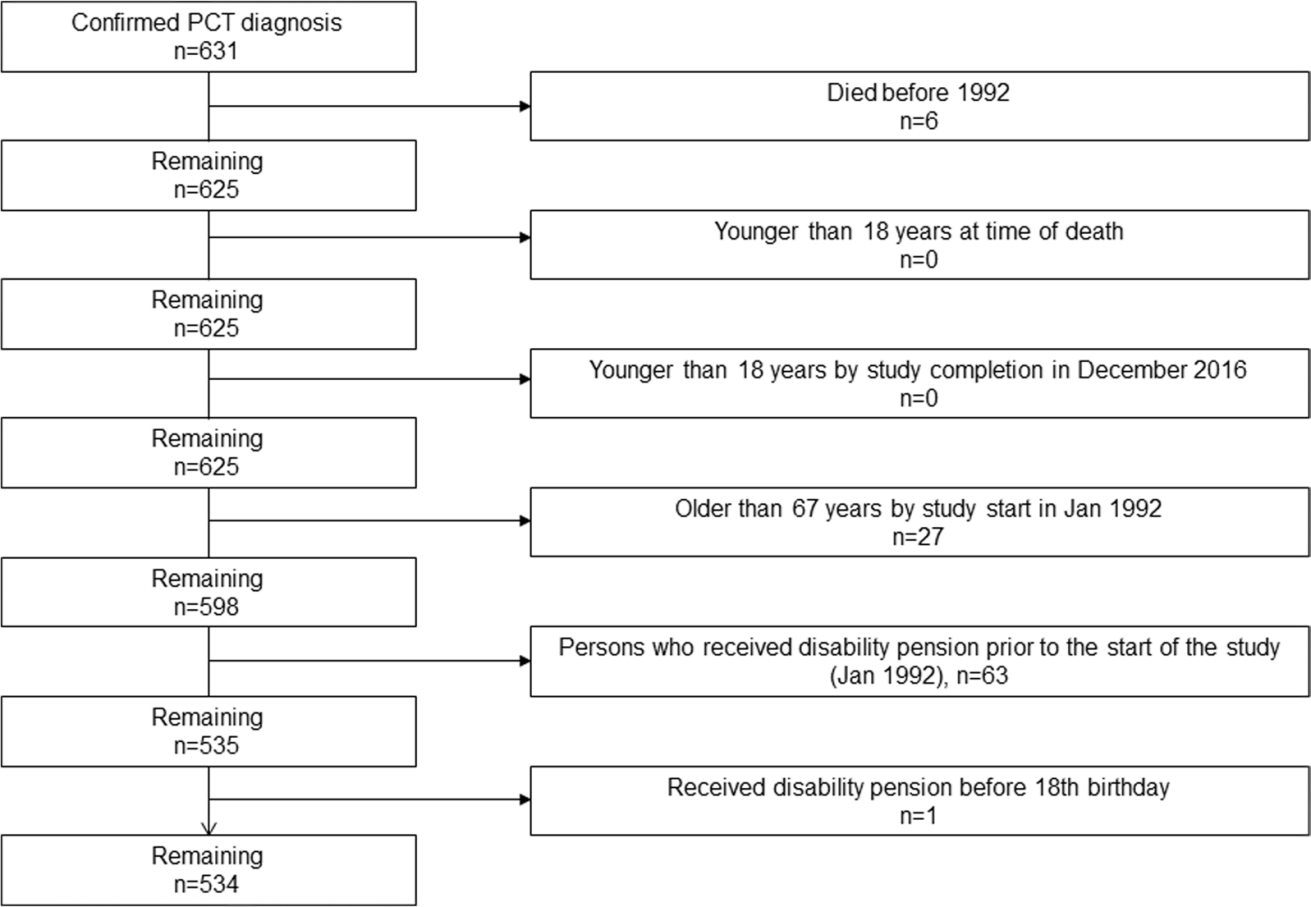
Overview of eligibility criteria and study sample

We conducted a population based, nationwide, matched-cohort study using registry data. Data regarding the exposure (PCT diagnosis) was collected previously to—and independent of—the outcome measures (i.e., LTSL, disability pension). The study period was from January 1992 to December 2016. Of the 811 persons with a confirmed PCT diagnosis in Norway by December 2016, 631 persons participated in the current study (78%), of whom 534 were eligible (sporadic PCT, n = 228; familial PCT, n = 240, unclassified PCT, n = 66). The exclusion criteria are depicted in Fig. 1.

The reference population consisted of 4,951,586 eligible adults aged 18–67 years (i.e., working age in Norway). We compared each person with PCT to 40 randomly selected controls from the reference population, matched on exact year of birth, sex and educational attainment (no education, primary and middle school education (1–10 years of education), intermediate education (11, 12, 13 years of education), tertiary education (14 years or more of education), and unspecified). This resulted in 21,360 controls.

Porphyrin analyses of urine, faeces and plasma, required to establish the PCT diagnosis, were conducted at the Department of Medical Biochemistry and Pharmacology, Haukeland University Hospital [3], using algorithms described by Badminton and colleagues [28]. Sequencing of the *UROD* gene was performed at the Center for Medical Genetics and Molecular Medicine at Haukeland University Hospital to establish if the patient had familial or sporadic PCT. Where DNA analysis had not been performed, participants were registered as unclassified PCT.

### Statistical analyses

Annual incidence rates by person/years on study and hazard ratios, calculated using Cox proportional regression models, were used to assess if persons with PCT had an increased risk of first LTSL or disability pension. A PCT diagnosis (yes/no) or PCT-subtype (familial PCT, sporadic PCT, unclassified PCT) were the exposure variables, and first ever LTSL and disability pension (yes/no) were the outcome variables. Age at event was the timescale and we censored for time of death and when assessing LTSL, time of disability pension. Persons were enrolled into the study in 1992 or when turning 18 years, and exited at the retirement age of 67 years, death, the event of interest, or study termination in 2016—whichever occurred first. Risk was assessed over the entire working-age, rather than after diagnosis, to better describe the life course of these patients and indirect effects of PCT related risk factors and comorbidities on the outcomes. We calculated Kaplan–Meier curves to plot increased risk over age. To investigate if persons with PCT accessed LTSL more frequently and for a longer duration, we explored differences to the matched controls regarding the count of total days and episodes of LTSL, divided by person-years. Predicted counts, incidence-rate ratios (IRRs) and 95% confidence intervals (CI) of annual LTSL days and episodes were calculated by zero-inflated negative binominal regression models with robust standard errors. The models are appropriate for over dispersed data with excess zeros (i.e., no LTSL events over follow-up) [29]. Day one of LTSL was counted from the 17th day of consecutive sick leave absences. Separate models were constructed for PCT overall and the sub-groups. The proportionality assumption of the Cox models was assessed by inspecting Kaplan–Meier curves and the log(− log(survival)) versus log(time) graphs for fixed covariates, including time-dependent covariates in the model for all covariates, and tests of the non-zero slope. No major violations were detected.

To investigate the temporal relationship between risk of LTSL and disability pension with PCT, we conducted separate Cox regression models and zero-inflated negative binominal models with robust standard errors for the time before, during and following a PCT diagnosis. This was defined accounting for likely diagnostic delay and time from diagnosis to full clinical and biochemical remission in the majority of patients. ‘Pre-PCT’ included all events registered prior to 2 years before the diagnosis; ‘during-PCT’ referred to the two immediate years prior to and following the PCT diagnosis (i.e., a 4 years period); ‘post-PCT’ was classified as events occurring later than 2 years after the diagnosis was established. Person-time was defined separately for the three time categories accounting for working-age range (18–67 years), study period (January 1992–December 2016), the event of interest, retirement and death, or whichever came first. When assessing risk in the period before, during and after a PCT diagnosis, the 40 controls were matched based on age in years at time of porphyria diagnosis, rather than age at study start. Sex and educational attainment were still accounted for in this matching.

The clinical and biochemical differences between persons with PCT who accessed disability pension to those who did not were assessed by Chi-square for categorical outcome variables and independent samples t-tests for continuous based outcome variables. To assess risk of specific diagnostic codes of interest for LTSL and disability pension, we calculated IRRs and CIs using Poisson regression with robust standard errors and offset for months on study. Not all disability pension events had a corresponding diagnostic code (missing data for entire population = 8.9%; missing for PCT cases = 3.4%).

We conducted a crude sensitivity analysis of the main analyses to investigate the potential effect of consent bias. The analysis included all cases known to have PCT in Norway aged between 18 and 67 years to December 2016 (n = 721). The rate of new LTSL or disability pension cases was based on the same rate observed in matched controls (i.e., assuming persons who did not consent did not vary from the population). We then calculated a crude relative risk point estimate (crude in that it does not account for person-years, censoring or differences in sex, age or educational attainment in persons who did not consent) and CIs using a subtraction method [30].

Stata&SE Version 16 for Windows was used for all statistical analyses (StataCorp Stata Statistical, Software, College Station, TX, USA).

### Ethical approval

The study was approved by the Western Regional Committee for Medical and Health Research Ethics, Norway (reference number: 2012/753).

## Results

### Baseline characteristics

Persons with sporadic and familial PCT did not differ from the general population in relation to sex ratio, although all PCT sub-groups were significantly older at the study start (Table 1). Persons with sporadic and unclassified PCT generally had less acquired years of formal education than persons with familial PCT or the general population (Table 1).

**Table 1:** Personal characteristics of participants with sporadic, familial and unclassified PCT, compared to matched controls and the total population for the main observed covariates (18–67 years of age, years 1992–2016)

Characteristics	Sporadic PCT (n = 228)		Matched cohort (n = 9120)		Familial PCT (n = 240)		Matched cohort (n = 9600)		Unclassified PCT (n = 66)		Matched cohort (n = 2640)		Population (n = 4,951,586)	
	n/ mean	%/ (SD)	n/ mean	%/ (SD)	n/ mean	%/ (SD)	n/ mean	%/ (SD)	n/ mean	%/ (SD)	n/ mean	%/ (SD)	n/ mean	%/ (SD)
Mean follow-up time (years)	18.4	6.3			20.1	6.2			16.4	6.8				
Sex—male	114	50.0	114	50.0	116	48.3	4640	48.3	45	68.2	1800	68.2	2,557,200	51.6
Age at study start—years	42.6	(9.91)	42.6	(9.91)	38.4	(13.1)	38.4	(13.1)	45.8	(11.5)	45.8	(11.5)	29.2	(13.6)
*Highest level of education attained*														
Unspecified	3	1.3	120	1.3	2	0.8	80	0.8	0	0.0	0	0.0	631,263	12.8
Primary/middle education (1–10 yrs)	76	33.3	3040	33.3	46	19.2	1840	19.2	23	34.9	920	34.9	1,049,128	21.2
Intermediate education (11–13 years)	114	50.0	4560	50.0	118	49.2	4720	49.2	30	44.5	1200	44.5	1,827,258	36.9
Tertiary education (14+ years)	35	15.4	1400	15.4	74	30.8	2960	30.8	13	19.7	520	19.7	1,443,937	29.2

Mean person-years calculated from age at entry into study (January 1992 or when 18th birthday) to age at exit from study (disability, retirement at age 67 years, study end in December 2016, or death—whichever came first)

### Risk of first ever LTSL and disability pension

**Fig. 2:**
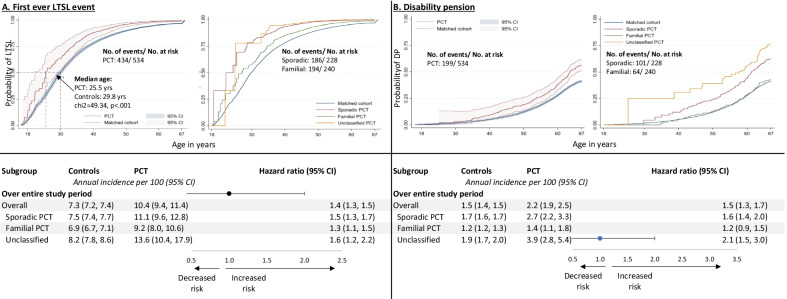
Kaplan–Meier estimates and subgroup analyses of long-term sick leave (LTSL) and disability pension

80.9% of persons with PCT compared to 71.0% of the matched controls accessed LTSL over 211,784.8 person-years at risk under observation. The annual incidence was 10.4% for persons with PCT compared to 7.3% in matched controls, constituting a 1.4-fold increased risk (HR = 1.4, CI 1.3, 1.5) (Fig. 2). Risk was greatest in persons with sporadic (HR = 1.5, CI 1.3, 1.7) and unclassified (HR = 1.6, CI 1.2, 2.2) PCT, but also increased in persons with familial PCT (HR = 1.3, CI 1.2, 2.2) (Fig. 2).

37.3% of persons with PCT accessed disability pension compared to 26.6% of matched controls resulting in a 1.5-fold (HR = 1.5, CI 1.3, 1.7) increased risk (Fig. 2). Within subgroups, risk was increased 1.6 (HR = 1.6, CI 1.4, 2.0) in persons with sporadic PCT, 2.1 (HR = 2.1, CI 1.5, 3.0) in persons with unclassified PCT and 1.2 (HR = 1.2, CI 0.9, 1.5) in persons with familial PCT (Fig. 2).

Excess risk of LTSL increased from 1.3 pre-PCT (HR = 1.3, CI 1.1, 1.4) to 1.5 during-PCT (HR = 1.5, CI 1.1, 2.1), but then decreased post-PCT (HR = 0.7, CI 0.5, 1.1). Excess risk of disability pension also increased from 1.3 pre-PCT (HR = 1.3, CI 1.0, 1.6) to 1.5 during-PCT (HR = 1.5, CI 1.0, 2.2), and increased to 2.0 post-PCT (HR = 2.0-fold, CI 1.5, 2.6). However, overlapping CIs suggest that these differences between pre-PCT, during-PCT and post-PCT were non-significant (Additional file 2: Table S2).

We found no evidence that effect of PCT on LTSL differed between men and women (*p* value for interaction sex#exposure = 0.636). There was a trend, although with considerable uncertainty, that the effect of PCT on LTSL was stronger with higher education (primary education [HR = 1.3, CI 1.1, 1.6], upper secondary school [HR = 1.4, CI 1.2, 1.6] and university education [HR = 1.5, CI 1.2, 1.8]). However, this was not a statistically significant interaction (*p* value for interaction education#exposure = 0.420).

### Sensitivity analysis of non-consent bias for main analyses

Including persons who did not consent with a known diagnosis of PCT in Norway (n = 187) with no additional risk of LTSL or disability as matched controls in a crude sensitivity analysis, would result in a relative risk estimate of 1.1 (CI 1.1, 1.2) for LTSL and 1.3 (CI 1.2, 1.4) for disability pension. Therefore, if persons who did not consent had, hypothetically, similar rates as matched controls, rather than persons with PCT who did consent, this would have reduced the effect sizes by 20% for LTSL and 30% for disability pension. The findings would, however, remain statistically significant.

### Days on sick leave and frequency of episodes

Overall, persons with PCT were on LTSL 1.3 more days per year (IRR = 1.3, CI = 1.0, 1.5) and had 1.1 more annual episodes than matched controls (IRR = 1.1, CI = 1.0, 1.5) (Table 2). Excess LTSL days increased from pre-PCT (IRR = 1.4, CI = 1.2, 1.7) to during PCT (IRR = 2.0, CI = 1.4, 3.0), but were reduced post-PCT (IRR = 0.30, CI = 0.1, 0.5) (Table 2). Excess LTSL events decreased from pre-PCT (IRR = 1.4, CI = 1.2, 1.7) to during-PCT (HR = 1.3, CI = 0.9, 1.8), and again in post-PCT (HR = 0.3, CI = 0.1, 0.5) (Table 2). Overlapping CIs suggest, however, that differences between pre and during—PCT estimates for events and total days were non-significant. Persons with sporadic disease had an increase of annual LTSL days and events overall, pre-PCT and during-PCT. This trend was less clear in persons with familial PCT (Table 2).

**Table 2:** Days on sick leave and frequency of episodes—by years on study and days on sick leave per episode

Social benefit type/group	Total number at risk	Predicted count divided by years on study (95% CI)	Yearly difference (95% CI)	Incident rate ratio (95% CI)
*Annual LTSL days*				
Reference	21,360	23.7 (22.7, 24.7)	0.0	1.0
PCT (total)	534	30.7 (26.6, 34.9)	7.0 (2.7, 11.3)	1.3 (1.1, 1.5)
Reference	9120	25.8 (24.3, 27.3)	0.0	1.0
Sporadic PCT	228	36.7 (29.8, 43.5)	10.9 (3.8, 17.9)	1.4 (1.2, 1.7)
Reference	9600	20.9 (19.8, 22.0)	0.0	1.0
Familial PCT	240	23.7 (18.0, 29.4)	2.8 (− 3.0, 8.6)	1.1 (0.9, 1.5)
Reference	2640	26.9 (22.6, 31.2)	0.0	1.0
Unclassified	66	35.8 (25.1, 46.4)	8.9 (− 2.6, 20.3)	1.3 (0.9, 1.9)
**Pre-PCT**				
Reference	19,359	24.8 (24.1, 25.4)	0.0	1.0
PCT (total)	484	35.7 (30.1, 41.4)	11.0 (5.3, 16.6)	1.4 (1.2, 1.7)
Reference	8884	23.4 (22.7, 24.2)	0.0	1.0
Sporadic PCT	222	34.7 (28.4, 41.1)	11.3 (4.9, 17.7)	1.5 (1.2, 1.8)
Reference	8280	25.1 (24.1, 26.2)	0.0	1.0
Familial PCT	207	31.0 (23.7, 38.3)	5.9 (− 1.5, 13.3)	1.2 (1.0, 1.6)
Reference	2195	28.7 (26.0, 31.3)	0.0	1.0
Unclassified	55	57.6 (25.7, 89.4)	28.9 (5.2, 61.3)	2.0 (1.1, 3.5)
**During-PCT**				
Matched cohort	15,916	6.1 (5.6, 6.6)	0.0	1.0
PCT (total)	392	12.4 (7.8, 16.9)	6.3 (1.7, 10.8)	2.0 (1.4, 3.0)
Reference	6060	4.7 (4.0, 5.3)	0.0	1.0
Sporadic PCT	142	12.5 (5.1, 19.8)	7.8 (0.3, 15.2)	2.7 (1.4, 4.9)
Reference	7735	6.2 (5.5, 6.9)	0.0	1.0
Familial PCT	201	10.2 (4.4, 16.1)	4.0 (− 1.8, 9.9)	1.7 (0.9, 3.0)
Reference	2121	9.9 (8.2, 11.5)	0.0	1.0
Unclassified	49	20.9 (3.8 (3.8, 37.9)	11.0 (− 6.1, 28.1)	2.1 (0.9, 4.9)
**Post-PCT**				
Matched cohort	13,868	15.9 (11.2, 20.6)	0.0	1.0
PCT (total)	335	4.4 (1.9, 6.8)	− 11.6 (− 16.8, − 6.4)	0.3 (0.1, 0.5)
Reference	5102	3.7 (3.1, 4.4)	0.0	1.0
Sporadic PCT	115	1.5 (− 1.1, 4.0)	− 2.3 (− 4.9, 0.4)	0.4 (0.1, 2.3)
Reference	6969	16.7 (10.1, 23.3)	0.0	1.0
Familial PCT	100	4.5 (1.0, 8.0)	− 12.2 (− 19.7, − 4.7)	0.3 (0.1, 0.6)
Reference	1797	47.4 (22.3, 72.5)	0.0	1.0
Unclassified	40	12.3 (1.4, 23.2)	− 35.1 (− 62.4, − 7.8)	0.3 (0.1, 0.7)
*Annual LTSL episodes*				
Matched controls	21,360	0.30 (0.29, 0.31)	0.0	1.0
PCT (total)	534	0.34 (0.30, 0.38)	0.04 (− 0.01, 0.08)	1.1 (1.0, 1.3)
Matched controls	9120	0.31 (0.30, 0.33)	0.0	1.0
Sporadic PCT	228	0.39 (0.30, 0.33)	0.07 (0.00, 0.15)	1.2 (1.0, 1.5)
Matched controls	9600	0.27 (0.25, 0.28)	0.0	1.0
Familial PCT	240	0.26 (0.21, 0.31)	0.01 (− 0.49, 0.48)	1.0 (0.8, 1.2)
Matched controls	2640	0.38 (0.34, 0.43)	0.0	1.0
Unclassified	66	0.41 (0.31, 0.51)	0.3 (− 0.08, 0.13)	1.1 (0.8, 1.4)
**Pre-PCT**				
Reference	19,359	0.24 (0.23, 0.24)	0.0	1.0
PCT (total)	484	0.33 (0.27, 0.39)	0.02 (− 0.01, 1.05)	1.4 (1.2, 1.7)
Reference	8884	0.24 (0.23, 0.25)	0.0	1.0
Sporadic PCT	222	0.34 (0.26, 0.41)	0.10 (0.03, 0.17)	1.4 (1.1, 1.8)
Reference	8280	0.23 (0.22, 0.24)	0.0	1.0
Familial PCT	207	0.29 (0.20, 0.38)	0.06 (− 0.03, 0.15)	1.3 (0.9, 1.7)
Reference	2195	0.25 (0.22, 0.27)	0.0	1.0
Unclassified	55	0.42 (0.19, 0.66)	0.17 (− 0.06, 0.41)	1.7 (1.0, 3.0)
**During-PCT**				
Matched cohort	15,916	0.07 (0.06, 0.08)	0.0	1.0
PCT (total)	392	0.09 (0.06, 0.12)	0.02 (− 0.01, 1.05)	1.3 (0.9, 1.8)
Reference	6060	0.04 (0.04, 0.05)	0.0	1.0
Sporadic PCT	142	0.08 (0.03, 0.13)	0.04 (− 0.01, 0.08)	1.8 (1.0, 3.3)
Reference	7735	0.07 (0.06, 0.08)	0.0	1.0
Familial PCT	201	0.07 (0.03, 0.11)	0.00 (− 0.04, 0.04)	1.0 (0.6, 1.8)
Reference	2121	0.14 (0.11, 0.17)	0.0	1.0
Unclassified	49	0.19 (0.05, 0.34)	0.05 (− 0.09, 0.20)	1.4 (0.6, 3.0)
**After-PCT**				
Matched cohort	13,868	0.17 (0.11, 0.22)	0.0	1.0
PCT (total)	335	0.04 (0.01, 0.07)	− 0.12 (− 0.18, − 0.06)	0.3 (0.1, 0.5)
Reference	5102	0.04 (0.03, 0.05)	0.0	1.0
Sporadic PCT	115	0.01 (0.00, 0.02)	− 0.03 (− 0.05, − 0.02)	0.2 (0.0, 0.8)
Reference	6969	0.20 (0.11, 0.29)	0.0	1.0
Familial PCT	100	0.06 (0.01, 0.11)	− 0.14 (− 0.24, − 0.04)	0.3 (0.1, 0.7)
Reference	1797	0.39 (0.16, 0.62)	0.0	1.0
Unclassified	40	0.08 (0.00, 0.15)	− 0.32 (− 0.56, − 0.08)	0.2 (0.1, 0.6)

Models and predicted counts based on a zero-inflated negative binominal model with robust standard errors. Day one of long-term sick leave is counted from the 17th day of consecutive sick leave absences. Sick leave days and episodes were divided by person-years on study, either for the entire study period, 2 years or more before (pre-PCT), within 2 years (during-PCT) and more than 2 after (post-PCT)—the age at the porphyria diagnosis. Estimates include 0 counts (i.e., no sick leave episode during period of study). Persons who accessed disability pension, retired, or died before these time periods were excluded from the time at risk cases. LTSL,  long-term sick leave

### LTSL and disability pension causes

Compared to matched controls, LTSL risk was increased for general and unspecific symptoms in persons with familial PCT (IRR = 1.4, CI = 1.0, 2.0), digestive issues (IRR = 2.5, CI = 1.3, 4.8), and muscle and joint pain (IRR = 2.1, CI = 1.3, 3.3) in persons with sporadic PCT, and endocrine and metabolic disorders for both sporadic PCT (IRR = 13.3, CI = 9.5, 18.7), and familial PCT (IRR = 15.0, CI = 10.1, 22.3). Compared to matched controls, risk of disability pension in persons with PCT was increased due to malignant neoplasms (IRR = 2.4, CI = 1.4, 4.2), PCT (IRR = 49.2, CI = 38.8, 62.4), hereditary hemochromatosis (n = 2, IRR = 29.1, CI = 10.8, 78.8), cerebrovascular diseases (n = 7, IRR = 3.2, CI = 1.6, 6.7), substance and alcohol dependence (n = 7, IRR = 5.0, CI = 2.5, 10.1), neurotic and mood—disorders (n = 21, IRR = 1.7, CI = 1.1, 2.6), diseases of the skin and subcutaneous tissue (n = 4, IRR = 4.2, CI = 1.6, 11.0), and diseases of the musculoskeletal system & connective tissue (IRR = 2.5, CI = 1.9, 3.2). In the subgroup analysis, risk was only increased in persons with sporadic PCT for malignant neoplasms (IRR = 3.0, CI = 1.4, 6.2), and neurotic and mood disorders (IRR = 2.2, CI = 1.2, 3.9), and increased in both persons with sporadic PCT (IRR = 3.3, 2.4, 4.6) and familial PCT (IRR = 1.6, CI = 1.0, 2.6) for diseases of the musculoskeletal system and connective tissue. Prevalence rates and IRRs for LTSL and disability pension diagnostic codes are depicted in Tables 3 and 4, respectively.

**Table 3:** Diagnostic codes for long-term sick leave

Diagnosis (ICPC-2)	Cases (one count per person per diagnosis)	Crude prevalence per 100	Incidence rate ratios (95% CI)
**General and unspecific (ICPC2: A)**			
Reference (total)	19,215/21,360	10.0	1.0
PCT (total)	68/534	12.7	1.1 (0.9, 1.4)
Reference	877/9120	9.6	1.0
Sporadic PCT	34/228	14.9	1.4 (1.0, 2.0)
Reference	1065/9600	11.1	1.0
Familial PCT	31/240	12.9	1.0 (0.7, 1.5)
**Weakness (ICPC2: A04)**			
Reference (total)	809/21,360	3.8	1.0
PCT (total)	29/534	5.4	1.2 (0.8, 1.8)
Reference	305/9120	3.3	1.0
Sporadic PCT	11/217	4.8	1.2 (0.7, 2.2)
Reference	423/9600	4.4	1.0
Familial PCT	17/240	7.1	1.4 (0.9, 2.3)
**Digestive (ICPC2: D)**			
Reference (total)	280/21,360	1.3	1.0
PCT (total)	15/534	2.8	1.9 (1.1, 3.1)
Reference	120/9120	1.3	1.0
Sporadic PCT	9/217	4.0	2.5 (1.3, 4.8)
Reference	130/ 9600	1.4	1.0
Familial PCT	5/240	2.1	1.4 (0.6, 3.4)
**Hypertension/ elevated blood pressure (ICPC2: K85-K87)**			
Reference (total)	657/21,360	3.1	1.0
PCT (total)	14/534	2.6	0.8 (0.5, 1.3)
Reference	291/9120	3.2	1.0
Sporadic PCT	8/217	3.5	1.1 (0.5, 2.1)
Reference	289/9600	3.0	1.0
Familial PCT	3/240	1.3	0.2 (0.1, 1.2)
**Ischemic heart disease (ICPC2: K74)**			
Reference (total)	297/21,360	1.4	1.0
PCT (total)	3/534	0.6	0.4 (0.1, 1.4)
Reference	142/9120	1.6	1.0
Sporadic PCT	0/217	0.0	0.0 (0.0, 0.0)
Reference	113/9600	1.2	1.0
Familial PCT	1/240	0.4	0.4 (0.1, 2.6)
**Muscle sympt/pain, joint sympt/pain (ICPC2: L18,-L20)**			
Reference (total)	801/21,360	3.8	1.0
PCT (total)	31/534	5.8	1.4 (1.0, 2.1)
Reference	345/9120	3.8	1.0
Sporadic PCT	19/217	8.3	2.1 (1.3, 3.3)
Reference	376/9600	3.9	1.0
Familial PCT	11/240	4.6	1.1 (0.6, 2.0)
**Acute stress reaction (anxiety) (ICPC2: P02)**			
Reference (total)	1209/21,360	5.7	1.0
PCT (total)	33/534	6.2	0.9 (0.6, 1.2)
Reference	464/9120	5.1	1.0
Sporadic PCT	16/217	7.0	1.1 (0.7, 1.8)
Reference	642/9600	6.7	1.0
Familial PCT	14/240	5.8	0.7 (0.4, 1.2)
**Feeling depressed/depressive disorders (ICPC2: P03)**			
Reference (total)	2612/21,360	12.2	1.0
PCT (total)	73/534	13.7	1.0 (0.8, 1.3)
Reference	1074/9120	11.8	1.0
Sporadic PCT	35/217	15.4	1.2 (0.8, 1.7)
Reference	1277/9600	13.3	1.0
Familial PCT	34/240	14.2	1.0 (0.7, 1.4)
**Endocrine, metabolism, disorders (ICPC2:T99)**			
Reference (total)	87/21,360	0.4	1.0
PCT (total)	48/534	9.0	13.1 (10.2, 16.8)
Reference	25/9120	0.3	1.0
Sporadic PCT	18/217	7.9	15.0 (10.1, 22.3)
Reference	52/9600	0.5	1.0
Familial PCT	11/240	10.8	13.3 (9.5, 18.7)
**Urological disorder/symptoms (ICPC2: U14)**			
Reference (total)	386/21,360	1.8	1.0
PCT (total)	11/534	2.1	1.0 (0.6, 1.9)
Reference	173/9120	1.9	1.0
Sporadic PCT	6/217	2.6	1.3 (0.6, 2.8)
Reference	171/9600	1.8	1.0
Familial PCT	4/240	1.7	0.9 (0.3, 2.3)

Incident rate ratios calculated by Poisson regression with robust standard errors and offset for the natural log of months on study. Persons with unclassified PCT were removed due to small numbers. ICPC-2 = International Classification of Primary Care, 2nd edition. ICPC-2 codes reflect the diagnostic reason for long-term sick leave. Other diagnoses that were investigated, but had counts less than 5 included acute myocardial disease, n = 3 (ICPC2: K75); peripheral neuritis/ neuropathy, n = 1 (ICPC2: N94), feeling anxious/nervous/tense, n = 3 (ICPC2: P01); affective disorder, n = 4 (ICPC2: P73) anxiety disorder, n = 3 (ICPC2: P74), suicide attempt, n = 0 (ICPC2:P77), kidney symptom/ complaint, n = 0 (ICPC2: U14)

### Characteristics of disability pension receivers

Persons who accessed disability pension were more likely to have sporadic or unclassified PCT and less likely to have familial PCT, be female, were on average 4 years older, have lower educational attainment, die over the study period, have a liver disease or Type II diabetes and report sore/fragile skin as a symptom of their PCT (Additional file 3: Table S3). On the other hand, no significant differences were observed for self-reported alcohol consumption, smoking, BMI, frequency of C282Y homozygosity, oestrogen use, other symptoms of PCT or total porphyrins or uroporphyrins (Additional file 3: Table S3).

## Discussion

### Main findings

In a nationwide matched-cohort study with a 24-year follow-up period, we found that persons with PCT had a 1.4-fold increased risk of a LTSL event and a 1.5-fold increased risk of disability pension during their working life compared to controls. Risk of disability pension was increased 1.3-times pre-PCT, 1.5-times during-PCT and 2.0-times after-PCT. Persons with sporadic and unclassified PCT had the greatest increased risk for both LTSL and disability pension.

### Contribution and interpretation

To our knowledge, no previous study has investigated the association between PCT and LTSL or disability pension, which are good indicators of morbidity during the working age of a patient group compared to the general population. We expected that PCT in itself, and independent of comorbidities, may result in increased access to LTSL during symptom onset, treatment and through to remission, and indeed our findings support this hypothesis. However, we also found that risk of disability pension was increased both before and mostly following a PCT diagnosis.

Susceptibility factors of PCT include excessive alcohol intake, hepatitis B and C infection and human immunodeficiency virus, especially in persons with sporadic PCT. Therefore, our findings of an increased risk of LTSL and disability pension likely reflect, in part, the role of comorbidities. In Norway, diagnostic delay is typically no longer than one to two years. So increased rates of disability pension two years or more before a diagnosis is likely due to comorbidities. This was reinforced by our finding of an effect in persons with sporadic but not familial PCT two years or more before a PCT diagnosis, given as a subgroup they generally have less comorbidities than persons with sporadic PCT.

Although many persons with PCT are treated and managed successfully, a number of patients experience relapses, especially if not followed up adequately. Therefore, one explanation for the excess risk of disability pension following the typical period of remission, may be due to a subset of patients who experience ongoing relapses or for whom remission is not reached. A recent meta-analysis reported a relapse rate between 5 to 17 per 100 person-years after remission for PCT [12]. Some patients also report diffuse long-term morbidity and a range of health problems they relate to their PCT diagnosis, despite successful treatment [20]. A report by the Norwegian Porphyria Registry found that the proportion of PCT patients who attended their annual medical check-up and fulfilled the recommended procedures was 66% in 2017 and only 53% had performed assessment of their porphyrin levels [25]. Analysis of porphyrins in urine and/or plasma is important in PCT because porphyrin concentrations indicate whether the patient is in remission or should start treatment to avoid new symptoms.

Diagnostic reasons for LTSL were generally similar to matched controls, such as muscle and joint pain, anxiety and depression, and was only increased compared to controls for digestive symptoms/disorders, general and unspecific symptoms and muscle and joint pain as well as endocrine and metabolic disorders, which includes the diagnosis of PCT. This may be explained in part by PCT itself or treatment for PCT, such as digestive issues as a known side-effect of chloroquine, used for treatment of PCT or related to for example hepatitis C, iron overload or other co-morbidities. Diagnostic reasons for disability pension also included substance and alcohol dependence, further supporting the role of comorbid diseases. Persons with PCT also had an increased risk of disability pension due to malignant neoplasms and diseases of the musculoskeletal system and connective tissue. In a previous study of the same cohort, we found that PCT was also associated with risk of early death and risk of hepatocellular carcinoma and gallbladder and biliary tract cancer [17]. Only seven persons were placed on a disability pension due to their PCT itself and four persons due to diseases of the skin & subcutaneous tissue, which is likely related to their PCT.

We observed a reduced risk of LTSL after the PCT diagnosis. However, during this time-period most persons with PCT were censored from the analysis due to a very high proportion having already had a LTSL event, having transitioned to disability pension or turned 67 years of age, transitioning to an old age pension. Additionally, the small subset of persons who had never had a LTSL event before or during their PCT diagnosis may also have differed from persons with PCT in general and, for a variety of reasons, may be less inclined to take LTSL. Estimates derived after a PCT diagnosis for LTSL are, therefore, likely unreliable. It is probable that this was less of an issue for disability pension given this is a less common event which typically occurs later in life and therefore, a larger proportion of persons remained in the analysis following their PCT diagnosis.

### Strengths and limitations

Strengths of the current study included the prospective nation-wide cohort design with a 24-year study follow-up. Data for outcomes and cofactors were drawn from national compulsory registries or administrative databases. To account in part for residual confounding we matched persons with PCT to controls of the same age, sex and educational attainment randomly selected from the entire population. We further investigated differences between persons with sporadic PCT and familial PCT as an indication of confounding by comorbidities. We further adjusted for education as a proxy of socioeconomic position and lifestyle.

The diagnosis of PCT is in most patients established in the 5th or 6th decade of life [20]. Therefore, comparing this patient group to the entire population will inevitably include comparisons to larger proportions of younger people. Even after adjusting for age in our models, this may lead to residual confounding and bias our estimates. We therefore used a ratio of 40 controls per case to ensure exact matching and sufficient statistical power for our main analyses. The controls were selected at random from the target population, reducing potential selection bias.

A limitation of the current study was that we were unable to include all persons with a PCT diagnosis across Norway. Except for persons who were deceased at the design phase of the study, participation was consent based and persons who did not participate may differ to persons who did. However, the participation rate of all known persons in Norway with a confirmed diagnosis of overt PCT was relatively high at 77.8% and we were able to investigate for potential bias by conducting a crude sensitivity analysis. In this analysis we included all known eligible persons in the main analysis and assessed the effect on risk ratios by specifying, hypothetically, that the additional members had a similar rate to matched controls. We find it reassuring that although the sensitivity analysis decreased the effect size, it did not change the overall study findings.

Another limitation of the study was the lack of some potentially important confounders at the level of the population, such as alcohol use and liver disease. We used clinical questionnaires from the Norwegian Porphyria Registry for persons with PCT, which provided some indication of differences between our sub-groups and persons with PCT who accessed disability pension compared to those who did not. However, this data did contain some missing data and is subjective, and therefore, there is some potential for information bias, especially in regards to lifestyle items. Given that confounding by lifestyle factors is likely in the current study, we also adjusted for educational attainment as a proxy of socioeconomic position and lifestyle, which was available for both cases and controls. However, despite our best efforts and use of a matched control group, residual confounding remains a potential source of bias in the current study.

Lastly, welfare systems across the world vary considerably and the rates in LTSL and disability pension in Norway are likely to be different compared to other countries. As discussed previously, Norway has a generous welfare system in which all residents who are reduced in their capacity to work due to illness or disability have, in principle, fair access to social security. In countries where such systems may be more difficult to access, illness due to PCT and comorbidities may result in a loss of employment, earlier access to age-retirement pension, or persons may have to continue working despite their morbidities. LTSL and disability leave can be considered proxy measures of morbidity in this patient group, which is likely to be similar across countries regardless of the welfare system.

## Conclusion

We found that persons with PCT had an increased risk of LTSL and disability pension. The risk for LTSL was increased before and during the typical time-period from first symptom onset, through to a diagnosis, treatment and remission. Risk for disability pension was increased over the entire study period, but was greatest following the typical period of remission for a porphyria diagnosis. The risk for disability pension was generally greater for persons with sporadic PCT than familial PCT, supporting the role of comorbidities. Overall, these outcomes indicate increased morbidity amongst PCT patients and, consequently, a decreased participation in the workforce. This is likely due to a number of factors such as PCT comorbid diseases and associated precipitating factors, PCT related symptoms, treatment side-effects and relapse. This highlights the importance of providing adequate follow-up of patients for their PCT and the associated comorbidities, not only until clinical remission but following this period as well.

## Supplementary Information

**Additional file 1.** **Supplementary Table 1.** Diagnostic codes.

**Additional file 2.** **Supplementary Table 2.** Survival analysis comparing risk of first ever long-term sick leave (LTSL) and disability pension to 40 matched controls over the entire study period and subdivided in time before, during and after the age at porphyria diagnosis.

**Additional file 3.** **Supplementary Table 3.** Baseline biochemical, clinical & lifestyle characteristics of participants with PCT who accessed disability pension compared to those who did not.

## Data Availability

The data that support the findings of this study are available from Statistics Norway but restrictions apply to the availability of these data, which were used under license for the current study, and so are not publicly available. Data are however available from the authors upon reasonable request and with permission of Statistics Norway.

## References

[1] Puy H, Gouya L, Deybach JC (2010). Porphyrias. Lancet.

[2] Fontanellas A, Martinez-Fresno M, Garrido-Astray MC, Perucho T, Moran-Jimenez MJ, Garcia-Bravo M (2010). Smoking but not homozygosity for CYP1A2 g-163A allelic variant leads to earlier disease onset in patients with sporadic porphyria cutanea tarda. Exp Dermatol.

[3] Aarsand AK, Boman H, Sandberg S (2009). Familial and sporadic porphyria cutanea tarda: characterization and diagnostic strategies. Clin Chem.

[4] Elder G (2010). Porphyria: genetics—encyclopedia of life sciences (ELS).

[5] Elder GH (1999). Alcohol intake and porphyria cutanea tarda. Clin Dermatol.

[6] Fargion S, Fracanzani AL (2003). Prevalence of hepatitis C virus infection in porphyria cutanea tarda. J Hepatol.

[7] Rossmann-Ringdahl I, Olsson R (2005). Porphyria cutanea tarda in a Swedish population: risk factors and complications. Acta Derm Venereol.

[8] Sampietro M, Fiorelli G, Fargion S (1999). Iron overload in porphyria cutanea tarda. Haematologica.

[9] Badminton MN, Elder GH (2002). Management of acute and cutaneous porphyrias. Int J Clin Pract.

[10] Harper P, Wahlin S (2007). Treatment options in acute porphyria, porphyria cutanea tarda, and erythropoietic protoporphyria. Curr Treat Options Gastroenterol.

[11] Singal AK (2019). Porphyria cutanea tarda: recent update. Mol Genet Metab.

[12] Salameh H, Sarairah H, Rizwan M, Kuo YF, Anderson KE, Singal AK (2018). Relapse of porphyria cutanea tarda after treatment with phlebotomy or 4-aminoquinoline antimalarials: a meta-analysis. Br J Dermatol.

[13] Siersema PD, ten Kate FJ, Mulder PG, Wilson JH (1992). Hepatocellular carcinoma in porphyria cutanea tarda: frequency and factors related to its occurrence. Liver.

[14] Gisbert JP, Garcia-Buey L, Alonso A, Rubio S, Hernandez A, Pajares JM (2004). Hepatocellular carcinoma risk in patients with porphyria cutanea tarda. Eur J Gastroenterol Hepatol.

[15] Fracanzani AL, Taioli E, Sampietro M, Fatta E, Bertelli C, Fiorelli G (2001). Liver cancer risk is increased in patients with porphyria cutanea tarda in comparison to matched control patients with chronic liver disease. J Hepatol.

[16] Linet MS, Gridley G, Nyren O, Mellemkjaer L, Olsen JH, Keehn S (1999). Primary liver cancer, other malignancies, and mortality risks following porphyria: a cohort study in Denmark and Sweden. Am J Epidemiol.

[17] Baravelli CM, Sandberg S, Aarsand AK, Tollanes MC (2019). Porphyria cutanea tarda increases risk of hepatocellular carcinoma and premature death: a nationwide cohort study. Orphanet J Rare Dis.

[18] Munoz-Santos C, Guilabert A, Moreno N, Gimenez M, Darwich E, To-Figueras J (2011). The association between porphyria cutanea tarda and diabetes mellitus: analysis of a long-term follow-up cohort. Br J Dermatol.

[19] OCED. Sickness, disability and work: breaking the barriers; 2010.

[20] Andersen J, Nordin K, Sandberg S (2016). Illness perception and psychological distress in persons with porphyria cutanea tarda. Acta Derm Venereol.

[21] Statistics Norway. Forløpsdatabasen-Trygd 2002: Statistics Norway; 2002. https://www.ssb.no/sosiale-forhold-og-kriminalitet/artikler-og-publikasjoner/forlopsdatabasen-trygd.

[22] Rostad Å, Støle E, Villanger JH, Aarsand AK, Sandberg S. Norsk porfyriregister—plan for forbedringstiltak 2013/20142013. http://www.kvalitetsregistre.no/getfile.php/Norsk/%C3%85rsrapporter/%C3%85rsrapport%202012%20porfyri.pdf.

[23] Bergen H. Nasjonalt kompetansesenter for porfyrisykdommer (NAPOS); 2021. https://helse-bergen.no/nasjonalt-kompetansesenter-for-porfyrisykdommer-napos.

[24] Norsk porfyriregister|Nasjonalt servicemiljø for medisinske kvalitetsregistre. https://www.kvalitetsregistre.no/registers/norsk-porfyriregister.

[25] Enes ÅR, Villanger JH, Thomsen J, Strand MEH, Støle E, Aarsand AK, et al. Norsk porfyriregister: Årsrapport for 2019 med plan for forbedringstiltak Haukeland universitetssjukehus; 2019. https://www.kvalitetsregistre.no/sites/default/files/2021-02/A%CC%8Arsrapport%202019%20Norsk%20porfyriregister.pdf.

[26] The Norwegian Tax Administration. National Population Register, 2021. https://www.skatteetaten.no/en/person/national-registry/.

[27] Statistics Norway. Nasjonal utdanningsdatabase; 2020. https://www.ssb.no/data-til-forskning/utlan-av-data-til-forskere/variabellister/utdanning/nasjonal-utdanningsdatabase.

[28] Badminton M, Deacon A, Elder G, Burtis C, Aashwood E, Bruns D (2012). The porphyrias and other disorders of porphyrin metabolism. Tietz textbook of clinical chemistry and molecular diagnostics.

[29] Long JS, Freese J (2006). Regression models for categorical dependent variables using stata (Second Edition).

[30] Newcombe RG (1999). Re: "confidence limits made easy: interval estimation using a substitution method". Am J Epidemiol.

