# Correction: Porphyria cutanea tarda and patterns of long-term sick leave and disability pension: a 24-year nationwide matched-cohort study

**DOI:** 10.1186/s13023-022-02329-2

**Published:** 2022-05-04

**Authors:** Carl Michael Baravelli, Aasne Karine Aarsand, Sverre Sandberg, Mette Christophersen Tollånes

**Affiliations:** 1 Department of Medical Biochemistry and Pharmacology, Haukeland University Hospital, Norwegian Porphyria Centre (NAPOS), P. O. Box 1400, 5021 Bergen, Norway; 2 Department of Disease Burden, Norwegian Institute of Public Health, Bergen, Norway; 3 Norwegian Institute of Public Health, Bergen, Norway; 4 Norwegian Organisation for Quality Improvement of Laboratory Examinations (NOKLUS), Haraldsplass Deaconess Hospital, Bergen, Norway; 5 Department of Global Public Health and Primary Care, University of Bergen, Bergen, Norway

## Correction to: Orphanet Journal of Rare Diseases (2022) 17:72 https://doi.org/10.1186/s13023-022-02201-3

Following the publication of the original article [1] the authors informed us that the risk ratios and error bars were omitted from Fig. 2.

**Fig. 2:**
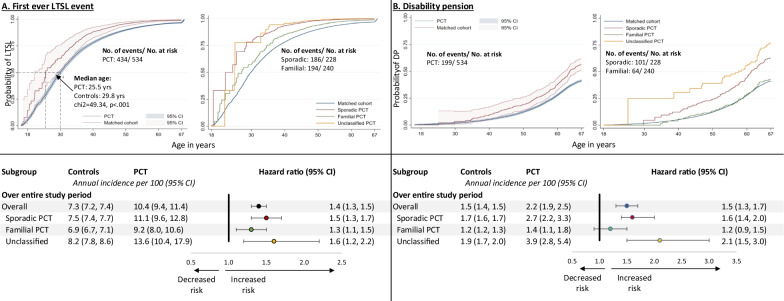
Kaplan–Meier estimates and subgroup analyses of long-term sick leave (LTSL) and disability pension

The correct Fig. 2 is included in this Correction and has already been updated in the original article.
