# Supplementary material for: Porphyria cutanea tarda and patterns of long-term sick leave and disability pension: a 24-year nationwide matched-cohort study
Source: Orphanet J Rare Dis. 2022 Feb 22;17:72. doi: 10.1186/s13023-022-02201-3 (PMC8862313; doi:10.1186/s13023-022-02201-3)
Supplement: Supplementary file 1 — Additional file 1. Supplementary Table 1. Diagnostic codes. [file 13023_2022_2201_MOESM1_ESM.docx]

Supplementary Table 1. Diagnostic codes

| **Long-term sick leave diagnoses** | **ICPC-2 code** |
| --- | --- |
| General and unspecified | A |
| Weakness/ tiredness general | A04 |
| Digestive | D01-D02, D06 |
| Cardiovascular | K |
| Acute myocardial disease | K75 |
| Ischemic heart disease | K76 |
| High blood pressure/hypertensive disorder | K85-K87 |
| Muscle/joint – pain/symptoms | L18-L20 |
| Peripheral neuritis/ neuropathy | N94 |
| Psychological | P01-P99 |
| Feeling anxious/nervous/tense | P01 |
| Acute stress reaction | P02 |
| Depressive disorder/ feeling depressed | P03 |
| Affective disorder | P73 |
| Anxiety disorder | P74 |
| Suicide attempt | P77 |
| Endocrine/ metabolism/ nutritional disorder, other | T99 |
| Kidney symptom/ complaint | U14 |
| **Disability pension diagnoses** | **ICD-10 / ICD-9 codes** |
| Neoplasms | C00-96, D45-47 / 140-239 |
| Diabetes mellitus | E10–14 / 250 |
| PCT | E80.1 / |
| Hereditary hemochromatosis | E83.1 / 275.03 |
| Ischemic heart disease | I20-I25 / 410-414 |
| Cerebrovascular diseases | I60-I69 / 430-438 |
| Chronic obstructive pulmonary disease | J43–44 / 496 |
| Substance and alcohol dependence | F10-19 / 303-305 |
| Neurotic and mood - disorders | F30-40/ 300, 308-309 |
| Diseases of the skin & subcutaneous tissue | L00-L99/ 680-709 |
| Diseases of the musculoskeletal system and connective tissue | M00-M99 / 710-739 |

Abbreviations: ICPC-2: International Classification of Primary Care – 2^nd^ edition; ICD-10: International Classification of Diseases (ICD) – and 10^th^ revision; ICD-9: International Classification of Diseases (ICD) – and 9^th^ revision.
