# Supplementary material for: Porphyria cutanea tarda and patterns of long-term sick leave and disability pension: a 24-year nationwide matched-cohort study
Source: Orphanet J Rare Dis. 2022 Feb 22;17:72. doi: 10.1186/s13023-022-02201-3 (PMC8862313; doi:10.1186/s13023-022-02201-3)
Supplement: Supplementary file 2 — Additional file 2. Supplementary Table 2. Survival analysis comparing risk of first ever long-term sick leave (LTSL) and disability pension to 40 matched controls over the entire study period and subdivided in time before, during and after the age at porphyria diagnosis. [file 13023_2022_2201_MOESM2_ESM.docx]

Supplementary Table 2. Survival analysis comparing risk of first ever long-term sick leave (LTSL) and disability pension to 40 matched controls over the entire study period and subdivided in time before, during and after the age at porphyria diagnosis

| **Social benefit type/ Group** |  | **Cases/ No. at risk (%)** |  | **Annual incidence per 100 (95% CI)** |  | **Matched hazard ratio (95%CI)** |
| --- | --- | --- | --- | --- | --- | --- |
| **First ever LTSL event** |  |  |  |  |  |  |
| **Pre-PCT** |  |  |  |  |  |  |
| Matched controls |  | 11,200/ 21,101 (53.1) |  | 8.3 (8.2, 8.5) |  | 1.0 |
| PCT (total) |  | 323/ 528 (61.2) |  | 10.8 (9.7, 12.0) |  | 1.3 (1.1, 1.4) |
| Matched controls |  | 5401/ 9120 (59.2) |  | 8.6 (8.4, 8.9) |  | 1.0 |
| Sporadic PCT |  | 154/ 228 (67.5) |  | 11.6 (9.9, 13.6) |  | 1.3 (1.1, 1.5) |
| Matched controls |  | 4828/ 9360 (51.6) |  | 7.8 (7.6, 8.0) |  | 1.0 |
| Familial PCT |  | 141/ 234 (39.7) |  | 9.6 (8.2, 11.4) |  | 1.2 (1.0, 1.5) |
| Matched controls |  | 9717 2621 (37.1) |  | 9.8 (9.2, 10.4) |  | 1.0 |
| Unclassified |  | 28/ 66 (42.4) |  | 14.1 (9.7, 20.4) |  | 1.4 (1.0, 2.0) |
| **During-PCT** |  |  |  |  |  |  |
| Matched controls |  | 1142/ 18480 (6.2) |  | 1.7 (1.6, 1.8) |  | 1.0 |
| PCT (total) |  | 42/ 462 (9.0) |  | 2.6 (1.9, 3.5) |  | 1.5 (1.1, 2.1) |
| Matched controls |  | 364/ 7400 (4.9) |  | 1.3 (1.2, 1.5) |  | 1.0 |
| Sporadic PCT |  | 15/ 185 (8.1) |  | 2.3 (1.4, 3.8) |  | 1.8 (1.1, 3.0) |
| Matched controls |  | 560/ 8720 (6.4) |  | 1.8 (1.6, 1.9) |  | 1.0 |
| Familial PCT |  | 18/ 218 (8.2) |  | 2.3 (1.4, 3.6) |  | 1.3 (0.8, 2.1) |
| Matched controls |  | 218/ 2360 (9.3) |  | 2.8 (2.4, 3.1) |  | 1.0 |
| Unclassified |  | 9/ 59 (15.3) |  | 4.8 (2.5, 9.2) |  | 1.7 (0.9, 3.4) |
| **Post-PCT** |  |  |  |  |  |  |
| Matched controls |  | 1355/ 14,000 (9.7) |  | 1.5 (1.5, 1.6) |  | 1.0 |
| PCT (total) |  | 23/ 350 (6.6) |  | 1.1 (0.7, 1.7) |  | 0.7 (0.5, 1.1) |
| Matched controls |  | 308/ 5440 (5.7) |  | 1.0 (0.9, 1.2) |  | 1.0 |
| Sporadic PCT |  | 2/ 136 (1.5) |  | 0.3 (0.1, 1.2) |  | 0.3 (0.1, 1.1) |
| Matched controls |  | 710/ 6640 (10.7) |  | 1.6 (1.5, 1.8) |  | 1.0 |
| Familial PCT |  | 16/ 166 (9.6) |  | 1.5 (0.9, 2.5) |  | 0.9 (0.6, 1.5) |
| Matched controls |  | 337/ 1920 (17.6) |  | 2.2 (2.0, 2.5) |  | 1.0 |
| Unclassified |  | 5/ 48 (10.4) |  | 1.4 (0.6, 3.4) |  | 0.6 (0.3, 1.5) |
| **Disability pension** |  |  |  |  |  |  |
| **Pre-PCT** |  |  |  |  |  |  |
| Matched controls |  | 2835/ 21,098 (13.4) |  | 1.3 (1.3, 1.4) |  | 1.0 |
| PCT (total) |  | 89/ 528 (16.9) |  | 1.6 (1.3, 2.0) |  | 1.3 (1.0, 1.6) |
| Matched controls |  | 1587/ 9120 (17.4) |  | 1.5 (1.5, 1.6) |  | 1.0 |
| Sporadic PCT |  | 55/ 228 (24.1) |  | 2.2 (1.7, 2.8) |  | 1.4 (1.1, 1.9) |
| Matched controls |  | 1010/ 9360 (10.8) |  | 1.0 (1.0, 1.1) |  | 1.0 |
| Familial PCT |  | 22/ 234 (9.4) |  | 0.9 (0.6, 1.3) |  | 0.8 (0.6, 1.3) |
| Matched controls |  | 238/ 2618 (9.0) |  | 1.6 (1.4, 1.8) |  | 1.0 |
| Unclassified |  | 12/ 66 (18.2) |  | 3.4 (1.9, 6.0) |  | 2.1 (1.2, 3.8) |
| **During-PCT** |  |  |  |  |  |  |
| Matched controls (10:1) |  | 703/ 18,445 (3.8) |  | 1.1 (1.0, 1.2) |  | 1.0 |
| PCT (total) |  | 26/ 461 (5.6) |  | 1.6 (1.1, 2.4) |  | 1.5 (1.0, 2.2) |
| Matched controls (10:1) |  | 329/ 7400 (4.5) |  | 1.2 (1.1, 1.4) |  | 1.0 |
| Sporadic PCT |  | 12/ 185 (6.5) |  | 1.8 (1.0, 3.2) |  | 1.5 (0.9, 2.7) |
| Matched controls (10:1) |  | 284/ 8685 (3.3) |  | 0.9 (0.8, 1.0) |  | 1.0 |
| Familial PCT |  | 9/ 217 (4.2) |  | 1.1 (0.6, 2.2) |  | 1.3 (0.7, 2.5) |
| Matched controls (10:1) |  | 90/ 2360 (3.8) |  | 1.2 (1.0, 1.5) |  | 1.0 |
| Unclassified |  | 5/ 59 (8.5) |  | 2.7 (1.1, 6.4) |  | 2.2 (0.9, 5.5) |
| **Post-PCT** |  |  |  |  |  |  |
| Matched controls (10:1) |  | 1244/ 13,960 (8.9) |  | 1.3 (1.2, 1.3) |  | 1.0 |
| PCT (total) |  | 56/ 349 (16.1) |  | 2.5 (1.9, 3.3) |  | 2.0 (1.5, 2.6) |
| Matched controls (10:1) |  | 403/ 5440 (7.4) |  | 1.3 (1.2, 1.4) |  | 1.0 |
| Sporadic PCT |  | 20/ 136 (14.7) |  | 2.9 (1.9, 4.5) |  | 2.4 (1.5, 3.7) |
| Matched controls (10:1) |  | 551/ 6600 (8.4) |  | 1.1 (1.0, 1.2) |  | 1.0 |
| Familial PCT |  | 21/ 165 (12.7) |  | 1.8 (1.2, 2.8) |  | 1.6 (1.0, 2.5) |
| Matched controls (10:1) |  | 290/ 1920 (15.1) |  | 1.7 (1.5, 1.9) |  | 1.0 |
| Unclassified |  | 15/48 (31.3) |  | 4.0 (2.4, 6.7) |  | 2.4 (1.4, 4.1) |

Controls randomly selected from the general population and matched exactly on age in years, sex and educational attainment. Hazard ratios calculated from Cox proportional regression models. Pre-PCT= time period. ‘Pre-PCT’ included all events registered prior to 2 years before the diagnosis; ‘during-PCT’ referred to the two immediate years prior to and following the PCT diagnosis; ‘post-PCT’ was classified as events occurring later than 2 years after the diagnosis was established.
