# Supplementary material for: Porphyria cutanea tarda and patterns of long-term sick leave and disability pension: a 24-year nationwide matched-cohort study
Source: Orphanet J Rare Dis. 2022 Feb 22;17:72. doi: 10.1186/s13023-022-02201-3 (PMC8862313; doi:10.1186/s13023-022-02201-3)
Supplement: Supplementary file 3 — Additional file 3. Supplementary Table 3. Baseline biochemical, clinical & lifestyle characteristics of participants with PCT who accessed disability pension compared to those who did not. [file 13023_2022_2201_MOESM3_ESM.docx]

Supplementary Table 3. Baseline biochemical, clinical & lifestyle characteristics of participants with PCT who accessed disability pension compared to those who did not

| **Characteristics** | **Disability pension (n=199)** | |  | **No disability pension (n=335)** | |  |  | |
| --- | --- | --- | --- | --- | --- | --- | --- | --- |
|  | **n** | **%** |  | **n/ mean** | **%/ (SD)** |  | **Chi^2^/ diff** | **p-value/ 95% CI_diff_** |
| **PCT sub-group** |  |  |  |  |  |  | 22.09 | .001 |
| Sporadic PCT | 101 | 50.8 |  | 127 | 37.9 |  |  |  |
| Familial PCT | 64 | 32.2 |  | 176 | 52.5 |  |  |  |
| Unclassified PCT | 34 | 17.1 |  | 32 | 9.6 |  |  |  |
| **Sex - male** | 90 | 45.2 |  | 109 | 54.8 |  | 5.00 | .025 |
| **Age at study start (1992) -** mean (SD) | 43.41 | (9.32) |  | 39.57 | (11.90) |  | 3.84 | (1.90, 5.78) |
| Age at PCT diagnosis  **-** mean (SD) |  |  |  |  |  |  |  |  |
| Age at disability pension  **-** mean (SD) |  |  |  | NA | NA |  | NA | NA |
| **Highest level of education attained** |  |  |  |  |  |  | 35.32 | .001 |
| Unspecified | 0 | 0.0 |  | 5 | 1.5 |  |  |  |
| Primary/middle edu (1-10 yrs) | 80 | 40.2 |  | 65 | 19.4 |  |  |  |
| Intermediate edu (11-13 yrs) | 92 | 46.2 |  | 170 | 50.8 |  |  |  |
| Tertiary edu (14+ yrs) | 27 | 13.6 |  | 95 | 28.4 |  |  |  |
| **Deceased over study period** | 52 | 26.1 |  | 44 | 13.1 |  | 14.30 | .001 |
| **Alcohol – amount of standard drinks per week** |  |  |  |  |  |  | 3.42 | .635 |
| <1 | 54 | 27.1 |  | 106 | 31.7 |  |  |  |
| 1-5 | 31 | 15.6 |  | 55 | 16.4 |  |  |  |
| 6-10 | 60 | 30.2 |  | 104 | 31.0 |  |  |  |
| >10 | 20 | 10.1 |  | 25 | 7.5 |  |  |  |
| Missing | 34 | 17.1 |  | 45 | 13.4 |  |  |  |
| **Tobacco: cigarettes** |  |  |  |  |  |  | 5.58 | .062 |
| Never / have quit | 61 | 30.7 |  | 125 | 37.3 |  |  |  |
| Occasionally / daily | 75 | 37.7 |  | 94 | 28.1 |  |  |  |
| Missing | 63 | 31.7 |  | 116 | 34.6 |  |  |  |
| **Body mass index (BMI)** | 26.08 | (4.24) |  | 26.00 | (3.79) |  | 0.09 | .844 |
| **Exogenerous triggers and co-morbid diseases** |  |  |  |  |  |  |  |  |
| High alcohol intake | 30 | 15.1 |  | 51 | 15.2 |  | 1.12 | .570 |
| Oestrogens | 28 | 14.1 |  | 50 | 14.9 |  | 1.11 | .574 |
| Liver disease | 21 | 10.6 |  | 15 | 4.5 |  | 9.47 | .009 |
| Haemochromatosis | 11 | 5.6 |  | 12 | 3.6 |  | 2.71 | .258 |
| Type II diabetes | 10 | 5.0 |  | 6 | 1.8 |  | 7.5 | .058 |
| Missing | 34 | 17.1 |  | 45 | 13.4 |  | 1.3 | .250 |
| **Haemochromatosis mutation (HFE)** |  |  |  |  |  |  |  |  |
| C282Y homozygozity | 9 | 4.5 |  | 26 | 7.8 |  | 2.14 | .144 |
| **PCT symptoms** |  |  |  |  |  |  |  |  |
| Vesicles | 136 | 68.3 |  | 215 | 64.2 |  | 4.92 | .085 |
| Hyperpigmentation | 122 | 61.3 |  | 197 | 58.8 |  | 2.76 | .251 |
| Painful fragile skin | 55 | 27.6 |  | 58 | 17.3 |  | 10.88 | .004 |
| Missing | 34 | 17.1 |  | 46 | 13.7 |  | 1.10 | .294 |
| **Biochemical characteristics** |  |  |  |  |  |  |  |  |
| Total porphyrins (mean, 95% CI), mnol creatinine | 1034.36 | 746.41 |  | 910.02 | (750.05) |  | 1.87 | (-6.57, 257.26) |
| Uroporphyrin (mean, 95% CI) | 703.83 | 538.76 |  | 659.78 | (581.66) |  | 44.05 | (-56.17, 144.26) |
| Heptaporphyrins (mean, 95% CI) | 250.79 | (176.39) |  | 207.28 | (155.66) |  | 43.51 | (14.51, 72.50) |
| **PCT decreased quality of life** |  |  |  |  |  |  | 3.46 | .177 |
| No | 57 | 28.6 |  | 118 | 35.2 |  |  |  |
| Yes | 75 | 37.7 |  | 103 | 30.8 |  |  |  |
| Missing | 67 | 33.7 |  | 114 | 34.0 |  |  |  |

Note: Uroporphyrins and heptaporphyrins expressed as nmol/mmol creatinine. Missing data from the clinical questionnaires from the Norwegian Porphyria Registry was around 17.1% in persons on disability pension and 13.4% for persons not on disability pension. There was no missing data for diagnostic, demographic or biochemical - data
